# Wealth's Opportunity

**Published:** 1913-05-17

**Authors:** 


### WEALTH'S OPPORTUNITY.

It is not often that wealthy people, whatever the amount of money they are prepared to expend, can secure an opportunity to invest a little of it so wisely and well as to produce nothing but good by its expenditure, not only to their fellows and to the world at large, but to themselves. One such opportunity, rare in the history of individual lives, is now offered to every man of substance in the City of London. Nowhere to an equal extent probably but in the City is there such high and continuous pressure upon the vital forces of individual workers who hold the most responsible and arduous positions in the financial and business world. For this reason there should be no class of men more fully alive to the importance of giving deliberately some of their substance, influence, and personal endeavour to the provision of the higher means of prevention and alleviation for the sick, whenever illness or sudden breakdown attacks the worker. The difficulty each relatively prosperous man in the City of London has to face in this connection is the power to concentrate with assured certainty upon an object, which will wholly fulfil the conditions we have just indicated. Such an object is offered to the intelligent wealthy throughout the City of London by the short statement issued by Guy's Hospital asking for £40,000. This money is required to complete the outlay on the new buildings of the medical school, and to make that school entirely self-supporting. The work of rebuilding is at an end, and the hospital at present possesses laboratories so complete and well equipped, with a residential college, library, pathological museum, and class rooms, that if the City will give this £40,000 the income from students' fees will be sufficient to maintain all departments as at present constituted, and to enable the school to carry on its twofold work of education and of research upon a self-supporting basis.

The best of City men may at first reply, What is this medical school to me? Everything, in the sense that its adequate equipment and maintenance upon a self-supporting basis means the maximum of efficiency and the best guarantees to every brain worker that the results of its work must continuously improve the chances of recovery and the prolongation of life to every man of this type. Guy's Hospital has a direct personal claim upon the intelligence and wealth of the City of London. In 1896, at the instigation of Mr. Cosmo Bonsor, the City joined hands with King Edward VII. (then Prince of Wales) and, in co-operation with the governors and medical staff of Guy's Hospital, raised for that institution at the dinner at the Imperial Institute on June 10 in that year the equivalent to nearly £170,000, which must go down to history as a maximum result from such a source. The encouragement thus given and the notice it attracted enabled the authorities of Guy's Hospital to place their finances upon the soundest possible basis. To-day, in pursuance of the wise and economical methods initiated by Mr. Bonsor, Lord Goschen, the present treasurer, with the governors, is wishful to crown the edifice by making the great medical school of Guy's Hospital self-supporting. To do this will secure for it the maximum of usefulness and efficiency. Forty thousand pounds seems a large sum, but as we have ourselves raised as much as £27,000 in the City in two days for pure charity, we know that it is certainly practicable to obtain the larger amount within a fortnight, especially as the money when given will contribute an assured safeguard to the continuous health and well-being of the most intellectual and hardest-worked men labouring within its boundaries.

On June 3 Mr. A. J. Balfour, member for the City of London, will inspect the new buildings and laboratories of the medical and dental school at Guy's and will open them. Is it too much to hope that the City will crown the splendid work they helped to accomplish in 1896 by making it their business to secure that £40,000 shall be placed in the hands of Lord Goschen as treasurer of Guy's Hospital by the evening of June 2 next? Every wealthy worker and thinker should rejoice to know that by contributing liberally to this £40,000, each donor will help to repay the debt of gratitude due to the medical staff of Guy's Hospital, who in 1896 made great sacrifices in order to guarantee the triumphant success of the grand efforts of that year.

